# Nutrient availability affects optimal growth strategy in predatory DPANN

**DOI:** 10.1128/msystems.01475-25

**Published:** 2025-11-18

**Authors:** Joshua N. Hamm

**Affiliations:** 1 Department of Marine Microbiology and Biogeochemistry, Royal Netherlands Institute for Sea Research 10209, Den Burg, the Netherlands; Florida Atlantic University, Boca Raton, Florida, USA

**Keywords:** archaea, symbiosis, predation, ecology

## Abstract

The DPANN archaea comprise a major microbial lineage that appears to be primarily host dependent. Despite the relative ubiquity of DPANN archaea across the biosphere, our understanding of their ecological role is limited due to the absence of cultivated representatives for most DPANN lineages. The majority of cultivated DPANN species are characterized as mildly parasitic ectosymbionts due to reliance on physical interactions with host cells. However, *Candidatus* Nanohaloarchaeum antarcticus has been reported to adopt a predatory lifestyle, resulting in the lysis of large numbers of host cells. The factors influencing DPANN-host interactions that drive *Ca*. Nha. antarcticus to adopt an aggressive lifestyle, although other DPANN appear not to, remain unclear. Here, I present a framework for understanding the ecological pressures specific to the *Ca*. Nha. antarcticus*–Halorubrum lacusprofundi* system and why a more aggressive, predatory lifestyle improves population persistence compared with a lifestyle more similar to other DPANN.

## PERSPECTIVE

The bacterial and archaeal domains each possess a major clade that appears to be predominantly host-associated (1–3). These two lineages, Patescibacteriota and DPANN archaea (named for the Diapherotrites, Parvarchaeota, Aenigmarchaeota, Nanoarchaeota, and Nanohaloarchaeota) (4), are highly diverse and abundant across the biosphere, but little is understood regarding their physiology and their contributions/impacts on the microbial communities they inhabit. This is in large part due to the small number of cultivated representatives for either group, which has limited our capacity to investigate their biology. As of the time of writing, there are only three DPANN archaeal lineages (Nanoarchaeota [5–7], Nanohaloarchaeota [8–10], and Micrarchaeota [11, 12]) and three Patescibacteriota lineages (Saccharibacteria [13–16], Paceibacteria [17, 18], and Gracilibacteria [19]) with cultivated representatives. The majority of DPANN and Patescibacteriota seem to adopt a mildly parasitic lifestyle with moderate negative fitness effects on host cells. However, recent reports indicate that a more aggressive predatory lifestyle is also adopted by some members of each lineage (16, 19–21).

Among the Patescibacteriota, several species have been reported to engage in predatory interactions with their hosts, resulting in host cell lysis (16, 19, 20, 22). Recently, the DPANN archaeon *Ca*. Nha. antarcticus was reported as the first DPANN known to trigger the lysis of its host species, *Hrr. lacusprofundi,* which it does at rates of up to 50% of host cells within 24 h (21). Other DPANN archaea have been reported to inhibit host proliferation, preventing host cells from dividing if too many parasite cells are attached, resulting in a loss of host cell viability (23). However, the aggression exhibited by *Ca*. Nha. antarcticus in inducing host cell lysis in laboratory cultures is orders of magnitude higher than these other DPANN archaea. A common question regarding the behavior of these predatory Patescibacteriota and DPANN archaea is “What is the advantage of killing the host?” The existence of other predatory microbes such as *Bdellovibrio bacteriovorous* (24) demonstrates that these strategies are viable across a broad range of environmental contexts. However, in the case of *Ca*. Nha. antarcticus, there are specific environmental factors at play that contribute to favoring an aggressive predatory lifestyle.

## ENVIRONMENTAL CONTEXT OF THE *CA.* NHA. ANTARCTICUS*–HRR. LACUSPROFUNDI* SYSTEM

*Ca*. Nha. antarcticus and *Hrr. lacusprofundi* are distributed across multiple hypersaline lakes in Antarctica (8), residing in systems with salt concentrations approaching saturation, bulk water temperatures of ~−15°C, and surface temperatures ranging from −16°C to 12°C (25–27). The best-characterized system in which this pair has been identified is that of Deep Lake, which currently holds the record for the least productive lake on the planet (26, 28). A major contributing factor in the low nutrient availability in this lake is the presence of only one primary producer in the system, the halophilic photosynthetic alga *Dunaliella salina* (28). The seasonal cycle of Antarctica, which consists of 6 months of constant sun exposure (summer), followed by 6 months of no sun exposure (winter), results in effectively no primary productivity for the lake for approximately half the year (25). Additionally, Antarctica does not have significant populations of animals that can provide nutrient input into these lakes. This is in direct contrast to more temperate hypersaline systems that experience sun exposure throughout the year and are often impacted by the activities of animals, including brine shrimp and migratory birds (29). These differences lead to temperate hypersaline systems having a more stable supply of nutrients across the annual cycle than systems found in the Antarctic. The low annual productivity of the Antarctic lakes results in slow growth of the microbial community, with proliferation rates estimated at ≤6 rounds per year (27). Despite this slow growth, the community is relatively abundant, indicating a capacity to persist through periods of low nutrient availability. It is therefore thought that proliferation of Deep Lake community members primarily takes place during summer, whereas during winter months, the community enters dormancy (25).

## INTERACTION DYNAMICS CHANGE AS A FUNCTION OF NUTRIENT AVAILABILITY

Cultivation of *Ca*. Nha. antarcticus has shown that the relative abundance of the nanohaloarchaeon in batch cultures increases with extended incubation times despite cultures undergoing starvation and overall biomass in the culture reducing (8). Microscopy of these extended incubations indicated that the majority of nanohaloarchaeal cells in these conditions are not attached to host cells (8). This contrasts with nanohaloarchaeal cells in exponential growth stages when the relative frequency of attachment events is much higher (8, 21). Genomic analyses and cryo-electron microscopy have indicated that the nanohaloarchaeon has the capacity to accumulate various types of nutrients, for example, in the form of starch and lipid droplets (8, 21). Taken together, these results suggest that *Ca*. Nha. antarcticus primarily attaches to and depredates its host during exponential growth, and during stationary/death phase, the nanohaloarchaeon instead remains detached and likely consumes the nutrient stores accumulated during exponential growth.

## AGGRESSION IS FAVORED DUE TO THE SEASONAL CYCLING OF NUTRIENT AVAILABILITY

The behavior of *Ca*. Nha. antarcticus in remaining detached during periods of low nutrient availability whilst growing aggressively during periods of nutrient excess, may have developed due to the Antarctic seasonal cycle. Despite differences in nutrient concentrations, there are parallels between laboratory culture growth stages and the summer and winter periods of the Antarctic cycle with regard to nutrient availability (nutrient excess, summer; nutrient limitation, winter). In other DPANN-host partnerships, host cells display characteristics of increased metabolic load and reduced capacity for proliferation (23). During the Antarctic winter months, when nutrients are limited, such an increase in metabolic demand on host cells could reduce host survivability and result in destabilization of both populations. It may therefore be more favorable for both organisms if the nanohaloarchaeon were to adopt a strategy of aggressive predation during periods of nutrient availability, that is, summer, and dormancy during periods of nutrient limitation, that is, winter.

**Fig 1:**
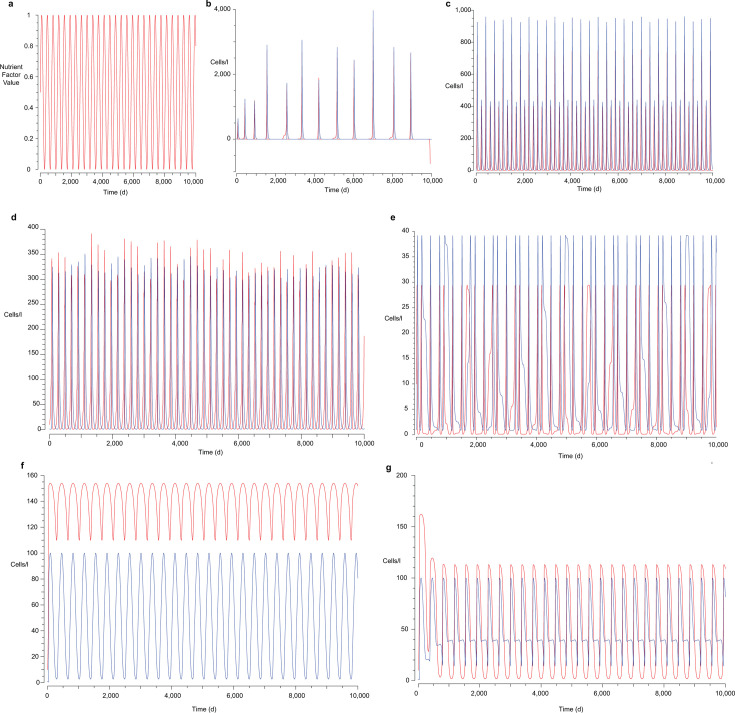
Modified Lotka-Volterra modeling of *Ca.* Nha. antarcticus–Hrr. lacusprofundi population dynamics. (**a**) Seasonal nutrient factor introduced to the Lotka-Volterra equations to link *Hrr.* lacusprofundi growth rates to seasonal availability of nutrients (represented as *F* in formulae). (**b–d**) Population dynamics of *Hrr*. lacusprofundi (red) and *Ca.* Nha. antarcticus (blue) predicted by the model when *Ca.* Nha. antarcticus continues to interact during periods of low nutrient availability (formulae 1a and 2a) with nutrient values ranging from either (**b and d**) 0–1 or (**c**) 0.5–1 and with a frequency of (**b and c**) 365 days or (**d**) 15 days, (**e**) Population dynamics of *Hrr.* lacusprofundi (red) and *Ca.* Nha. antarcticus (blue) when *Ca.* Nha. antarcticus avoids interactions during periods of low nutrient availability (formulae 1b and 2b). (**f**) Population dynamics of *Hrr.* lacusprofundi (red) and *Ca.* Nha. antarcticus (blue) when *Ca.* Nha. antarcticus avoids interactions during periods of low host proliferation (formulae 1b and 2c). (**g**) Population dynamics of *Hrr.* lacusprofundi (red) and *Ca.* Nha. antarcticus (blue) when *Ca.* Nha. antarcticus avoids interactions during periods of low host proliferation and nutrient availability (formulae 1b and 2d).

To demonstrate how nutrient availability impacts the long-term persistence of parasite and host populations, I modeled the interactions between *Ca*. Nha. antarcticus and *Hrr. lacusprofundi* using modified Lotka-Volterra equations (Table 1) with the COPASI software package (30). The standard Lotka-Volterra equation includes two formulae describing the rate of change for prey and predator populations as functions of the rate of proliferation of the prey, the rate of predation by the predator, and the rate of decay of the predator population. These Lotka-Volterra equations predict a cyclic relationship between predator-prey numbers. However, the standard Lotka-Volterra equations assume a constant rate of proliferation for prey species, which, under the environmental conditions in Deep Lake Antarctica, is unrealistic. Therefore, to model the difference between summer and winter productivity in the Antarctic lakes, I incorporated a nutrient availability factor with a regular wave function profile and a frequency corresponding to 365 days (Fig. 1a). I then factored this into the formulae for *Hrr. lacusprofundi* and *Ca*. Nha. antarcticus, resulting in a growth rate variable that trends toward zero during winter and which is maximal during summer.

**TABLE 1:** Details of formulae used to model population dynamics of *Hrr. lacusprofundi* and *Ca*. Nha. antarcticus^*a*^

Equation	Organism	Formula
1a	*Hrr. lacusprofundi*	$\frac{dH}{dt}=F\left(\alpha H\right)-\varepsilon NH$
1b	*Hrr. lacusprofundi*	$\frac{dH}{dt}=F\left(\alpha H-\varepsilon NH\right)$
2a	*Ca*. Nha. antarcticus	$\frac{dN}{dt}=\theta NH-\mu N$
2b	*Ca*. Nha. antarcticus	$\frac{dN}{dt}=F\left(\theta NH-\mu N\right)$
2c	*Ca*. Nha. antarcticus	$\frac{dN}{dt}=\frac{dH}{dt}\left(\theta NH-\mu N\right)$
2d	*Ca*. Nha. antarcticus	$\frac{dN}{dt}=F\frac{dH}{dt}\left(\theta NH-\mu N\right)$

^*a*^ *H* represents the number of *Halorubrum lacusprofundi* cells, *N* represents the number of *Ca*. Nha. antarcticus cells, *F* represents the nutrient factor (range: 0–1 or 0.5–1), α represents the rate of proliferation of *Halorubrum lacusprofundi* (0.1), ε represents the rate of predation of *Halorubrum lacusprofundi* by *Ca*. Nha. antarcticus (range: 0.0001–0.01), θ represents the rate of proliferation of *Ca*. Nha. antarcticus (range: 0.001–0.1), and μ is the specific decay rate of *Ca*. Nha. antarcticus (0.05).

I tested multiple scenarios with the inclusion of this nutrient factor in the model. In the first three, *Ca*. Nha. antarcticus continued to interact with *Hrr. lacusprofundi* cells, regardless of nutrient availability, but lysed host cells at 100-fold lower rates than other scenarios (Fig. 1b through d. In the others, *Ca*. Nha. antarcticus induced higher rates of lysis, but only during periods of nutrient availability and/or host proliferation (Fig. 1e through g). The addition of a seasonal cycle to the model results in the eventual collapse of both *Hrr. lacusprofundi* and *Ca*. Nha. antarcticus populations when the nanohaloarchaeon continues to engage in interactions with the host despite nutrient depletion (Fig. 1b). This holds true regardless of the relative rate at which *Ca*. Nha. antarcticus causes host cell lysis, with the only change being the number of seasonal cycles required for the system to collapse. Modification of the nutrient factor to increase the minimum (Fig. 1c: 0.5 ≤ F ≤ 1) or decrease the time for the cycle to complete (Fig. 1d, 15 days) restores persistence of the populations, indicating that collapse is specific to scenarios where nutrient availability approaches zero for extended lengths of time. In contrast, when predation was linked to nutrient availability (Fig. 1e), host proliferation rate (Fig. 1f), or a combination of both (Fig. 1g), the populations persisted indefinitely. As with the previous scenario, this result was robust regardless of the relative rates of lysis and proliferation, indicating the primary underlying cause of population collapse was the continued interaction of *Ca*. Nha. antarcticus with nutrient-deprived host cells. The model explains several observations from laboratory cultures, for example, the model predicts that after ~6 months with reduced nutrient availability, the abundance of nanohaloarchaeon and host should be approximately equal. This is in agreement with the results of cultivation efforts where extended incubations of up to 9 months yielded approximately equal population sizes of the two species (8). The model also predicts that transfer of cultures in the late-exponential phase would likely result in gradual dilution of the nanohaloarchaeon, as their relative abundance is low during this phase. This is again consistent with the results of cultivation, efforts where continuous transfer in the late-exponential phase (determined by OD_600_) resulted in loss of the nanohaloarchaeon from the culture (8).

**Fig 2:**
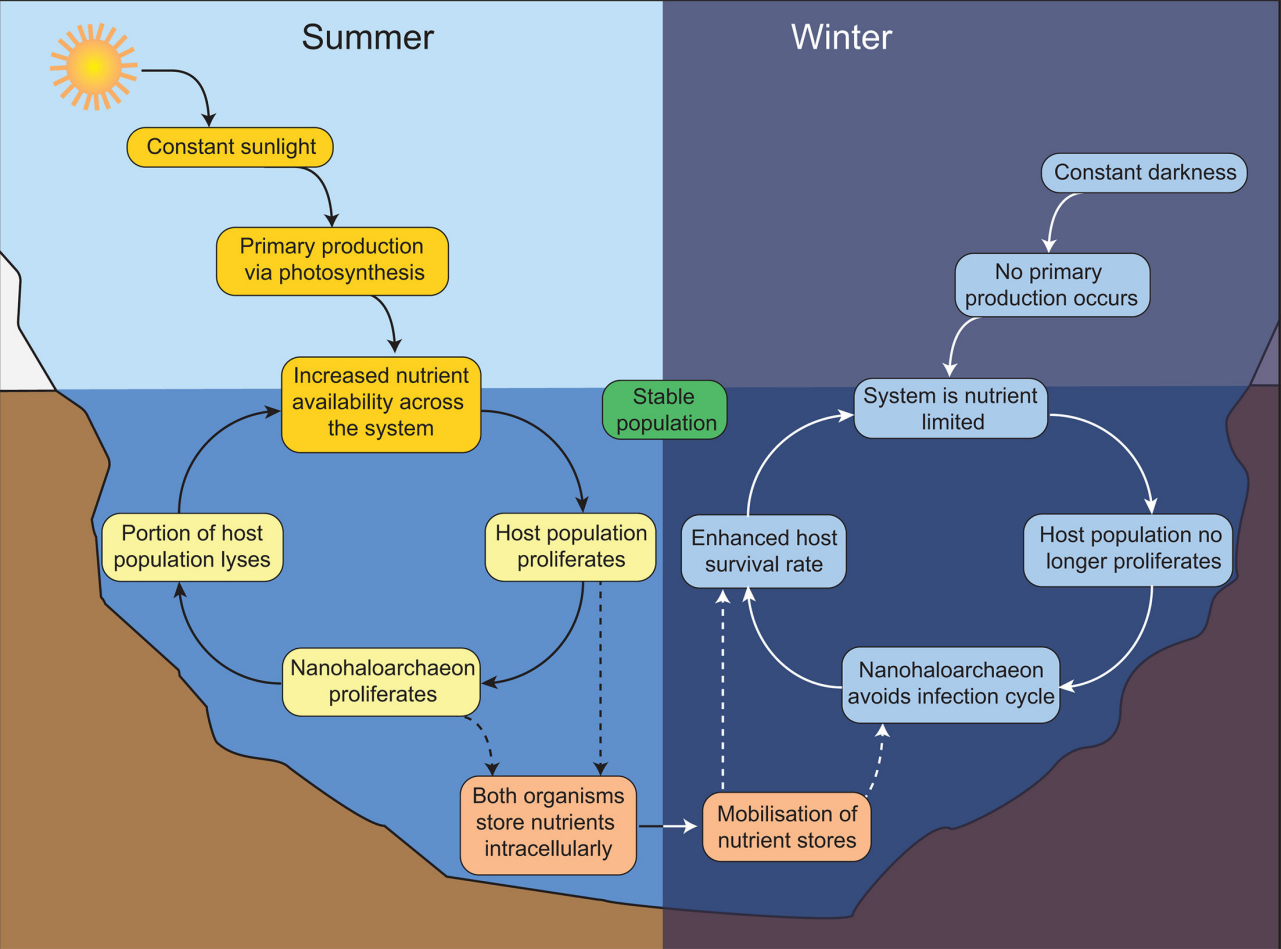
Concept diagram of the seasonal cycle of hypersaline lakes in Antarctica. The figure depicts the seasonal differences in the lakes *Ca.* Nha. antarcticus and *Hrr*. lacusprofundi inhabit and the corresponding effect on nutrient availability and behavior of the nanohaloarchaeon.

The predictions of the model indicate that increased aggression can result in a more stable seasonal cycle of both host and parasite populations if this behavior is modulated according to nutrient availability or host proliferation. This scenario yielded more persistent populations for both organisms and also brought the frequency of the predator-prey population cycle back to a period of approximately 1 year (Fig. 1e through g), in sync with the seasonal cycle. In contrast, less aggressive interactions that continue despite nutrient limitation were predicted to collapse both populations in the long term. By exploiting host populations during periods of nutrient availability, the actively replicating host population is likely more robust and better able to survive predation by the nanohaloarchaeon. The nanohaloarchaeal population can take advantage of this and accumulate sufficient nutrients to persist through the winter, allowing them to leave host cells undisturbed during nutrient-limited periods and thereby increase their probability of survival (conceptualized in Fig. 2). Increased aggression likely improves the speed at which nanohaloarchaeal cells accumulate necessary nutrients, although not significantly impacting persistence of the host population in the model. The tendency for other DPANN (including nanohaloarchaeota) to constantly associate with host cells may partially explain why *Ca*. Nha. antarcticus is the only detectable nanohaloarchaeon present in Antarctic hypersaline systems thus far (8). In contrast, the increased population sizes associated with constant associations in systems with more stable nutrient availability may explain the apparent predominance of these strategies in other cultivated DPANN.

## THE IMPORTANCE OF ECOLOGICAL CONTEXT

The case of *Ca*. Nha. antarcticus highlights that ecological context is critical in evaluating the optimal strategies for long-term population persistence of microbial parasites and their hosts. Although the behavior of *Ca*. Nha. antarcticus toward *Hrr. lacusprofundi* seems antagonistic, modeling predicts the strategy results in a healthier, more stable host population in the context in which this partnership evolved. These predictions are likely generally applicable to partnerships that evolve in ecosystems that experience long periods of nutrient depletion, whereas more consistent nutrient availability or more rapid flux allows for less aggressive partnerships to thrive. This emphasizes the importance of considering ecological context during the design of cultivation experiments, since maintenance of the *Hrr. lacusprofundi–Ca*. Nha. antarcticus partnership in a state of constant nutrient availability is not reflective of the environment that they inhabit. The ecological significance of observations made under such conditions must be tempered by this knowledge, or experiments should be designed to emulate the conditions these organisms experience in their native environment. It is only by considering discoveries from cultivation experiments alongside knowledge of ecological context that we can move toward a better understanding of microbial host-parasite interactions and their significance across the biosphere.

## References

[1] Castelle CJ, Banfield JF. 2018. Major new microbial groups expand diversity and alter our understanding of the tree of life. Cell 172:1181–1197. doi:10.1016/j.cell.2018.02.016 29522741

[2] Dombrowski N, Lee JH, Williams TA, Offre P, Spang A. 2019. Genomic diversity, lifestyles and evolutionary origins of DPANN archaea. FEMS Microbiol Lett 366:fnz008. doi:10.1093/femsle/fnz008 30629179 PMC6349945

[3] Castelle CJ, Brown CT, Anantharaman K, Probst AJ, Huang RH, Banfield JF. 2018. Biosynthetic capacity, metabolic variety and unusual biology in the CPR and DPANN radiations. Nat Rev Microbiol 16:629–645. doi:10.1038/s41579-018-0076-2 30181663

[4] Rinke C, Schwientek P, Sczyrba A, Ivanova NN, Anderson IJ, Cheng JF, Darling A, Malfatti S, Swan BK, Gies EA, Dodsworth JA, Hedlund BP, Tsiamis G, Sievert SM, Liu WT, Eisen JA, Hallam SJ, Kyrpides NC, Stepanauskas R, Rubin EM, Hugenholtz P, Woyke T. 2013. Insights into the phylogeny and coding potential of microbial dark matter. Nature 499:431–437. doi:10.1038/nature12352 23851394

[5] St. John E, Liu Y, Podar M, Stott MB, Meneghin J, Chen Z, Lagutin K, Mitchell K, Reysenbach A-L. 2019. A new symbiotic nanoarchaeote (Candidatus Nanoclepta minutus) and its host (Zestosphaera tikiterensis gen. nov., sp. nov.) from a New Zealand hot spring. Syst Appl Microbiol 42:94–106. doi:10.1016/j.syapm.2018.08.005 30195930

[6] Wurch L, Giannone RJ, Belisle BS, Swift C, Utturkar S, Hettich RL, Reysenbach AL, Podar M. 2016. Genomics-informed isolation and characterization of a symbiotic Nanoarchaeota system from a terrestrial geothermal environment. Nat Commun 7:12115. doi:10.1038/ncomms12115 27378076 PMC4935971

[7] Kato S, Ogasawara A, Itoh T, Sakai HD, Shimizu M, Yuki M, Kaneko M, Takashina T, Ohkuma M. 2022. Nanobdella aerobiophila gen. nov., sp. nov., a thermoacidophilic, obligate ectosymbiotic archaeon, and proposal of Nanobdellaceae fam. nov., Nanobdellales ord. nov. and Nanobdellia class. nov. Int J Syst Evol Microbiol 72:005489. doi:10.1099/ijsem.0.005489 35993221

[8] Hamm JN, Erdmann S, Eloe-Fadrosh EA, Angeloni A, Zhong L, Brownlee C, Williams TJ, Barton K, Carswell S, Smith MA, Brazendale S, Hancock AM, Allen MA, Raftery MJ, Cavicchioli R. 2019. Unexpected host dependency of Antarctic Nanohaloarchaeota. Proc Natl Acad Sci USA 116:14661–14670. doi:10.1073/pnas.1905179116 31253704 PMC6642349

[9] La Cono V, Messina E, Rohde M, Arcadi E, Ciordia S, Crisafi F, Denaro R, Ferrer M, Giuliano L, Golyshin PN, Golyshina OV, Hallsworth JE, La Spada G, Mena MC, Merkel AY, Shevchenko MA, Smedile F, Sorokin DY, Toshchakov SV, Yakimov MM. 2020. Symbiosis between nanohaloarchaeon and haloarchaeon is based on utilization of different polysaccharides. Proc Natl Acad Sci USA 117:20223–20234. doi:10.1073/pnas.2007232117 32759215 PMC7443923

[10] Reva O, Messina E, La Cono V, Crisafi F, Smedile F, La Spada G, Marturano L, Selivanova EA, Rohde M, Krupovic M, Yakimov MM. 2023. Functional diversity of nanohaloarchaea within xylan-degrading consortia. Front Microbiol 14:1182464. doi:10.3389/fmicb.2023.1182464 37323909 PMC10266531

[11] Golyshina OV, Toshchakov SV, Makarova KS, Gavrilov SN, Korzhenkov AA, La Cono V, Arcadi E, Nechitaylo TY, Ferrer M, Kublanov IV, Wolf YI, Yakimov MM, Golyshin PN. 2017. “ARMAN” archaea depend on association with euryarchaeal host in culture and in situ. Nat Commun 8:60. doi:10.1038/s41467-017-00104-7 28680072 PMC5498576

[12] Krause S, Gfrerer S, Reuse C, Dombrowski N, Villanueva L, Bunk B, Spröer C, Neu TR, Kuhlicke U, Schmidt-Hohagen K, Hiller K, Rachel R, Spang A, Gescher J. 2021. Unraveling the critical growth factors for stable cultivation of (nano-sized) Micrarchaeota. bioRxiv. doi:10.1101/2021.04.28.441856

[13] He X, McLean JS, Edlund A, Yooseph S, Hall AP, Liu S-Y, Dorrestein PC, Esquenazi E, Hunter RC, Cheng G, Nelson KE, Lux R, Shi W. 2015. Cultivation of a human-associated TM7 phylotype reveals a reduced genome and epibiotic parasitic lifestyle. Proc Natl Acad Sci USA 112:244–249. doi:10.1073/pnas.1419038112 25535390 PMC4291631

[14] Cross KL, Campbell JH, Balachandran M, Campbell AG, Cooper CJ, Griffen A, Heaton M, Joshi S, Klingeman D, Leys E, Yang Z, Parks JM, Podar M. 2019. Targeted isolation and cultivation of uncultivated bacteria by reverse genomics. Nat Biotechnol 37:1314–1321. doi:10.1038/s41587-019-0260-6 31570900 PMC6858544

[15] Xie B, Wang J, Nie Y, Tian J, Wang Z, Chen D, Hu B, Wu XL, Du W. 2022. Type IV pili trigger episymbiotic association of Saccharibacteria with its bacterial host. Proc Natl Acad Sci USA 119:e2215990119. doi:10.1073/pnas.2215990119 36454763 PMC9894109

[16] Batinovic S, Rose JJA, Ratcliffe J, Seviour RJ, Petrovski S. 2021. Cocultivation of an ultrasmall environmental parasitic bacterium with lytic ability against bacteria associated with wastewater foams. Nat Microbiol 6:703–711. doi:10.1038/s41564-021-00892-1 33927381

[17] Kuroda K, Kubota K, Kagemasa S, Nakai R, Hirakata Y, Yamamoto K, Nobu MK, Narihiro T. 2022. Novel cross-domain symbiosis between candidatus patescibacteria and hydrogenotrophic methanogenic archaea methanospirillum discovered in a methanogenic ecosystem. Microbes Environ 37:ME22063. doi:10.1264/jsme2.ME22063 36372432 PMC9763046

[18] Kuroda K, Yamamoto K, Nakai R, Hirakata Y, Kubota K, Nobu MK, Narihiro T. 2022. Symbiosis between Candidatus patescibacteria and archaea discovered in wastewater-treating bioreactors. mBio 13:e0171122. doi:10.1128/mbio.01711-22 36043790 PMC9600506

[19] Yakimov MM, Merkel AY, Gaisin VA, Pilhofer M, Messina E, Hallsworth JE, Klyukina AA, Tikhonova EN, Gorlenko VM. 2022. Cultivation of a vampire: “Candidatus Absconditicoccus praedator”. Environ Microbiol 24:30–49. doi:10.1111/1462-2920.15823 34750952

[20] Moreira D, Zivanovic Y, López-Archilla AI, Iniesto M, López-García P. 2021. Reductive evolution and unique predatory mode in the CPR bacterium Vampirococcus lugosii. Nat Commun 12:2454. doi:10.1038/s41467-021-22762-4 33911080 PMC8080830

[21] Hamm JN, Liao Y, von Kügelgen A, Dombrowski N, Landers E, Brownlee C, Johansson EMV, Whan RM, Baker MAB, Baum B, Bharat TAM, Duggin IG, Spang A, Cavicchioli R. 2024. The parasitic lifestyle of an archaeal symbiont. Nat Commun 15:6449. doi:10.1038/s41467-024-49962-y 39085207 PMC11291902

[22] Guerrero R, Pedros-Alio C, Esteve I, Mas J, Chase D, Margulis L. 1986. Predatory prokaryotes: predation and primary consumption evolved in bacteria. Proc Natl Acad Sci USA 83:2138–2142. doi:10.1073/pnas.83.7.2138 11542073 PMC323246

[23] Giannone RJ, Wurch LL, Heimerl T, Martin S, Yang ZM, Huber H, Rachel R, Hettich RL, Podar M. 2015. Life on the edge: functional genomic response of Ignicoccus hospitalis to the presence of Nanoarchaeum equitans. ISME J 9:101–114. doi:10.1038/ismej.2014.112 25012904 PMC4274422

[24] Ezzedine JA, Desdevises Y, Jacquet S. 2022. Bdellovibrio and like organisms: current understanding and knowledge gaps of the smallest cellular hunters of the microbial world. Crit Rev Microbiol 48:428–449. doi:10.1080/1040841X.2021.1979464 34595998

[25] Cavicchioli R. 2015. Microbial ecology of Antarctic aquatic systems. Nat Rev Microbiol 13:691–706. doi:10.1038/nrmicro3549 26456925

[26] Cavicchioli R. 2006. Cold-adapted archaea. Nat Rev Microbiol 4:331–343. doi:10.1038/nrmicro1390 16715049

[27] Williams TJ, Allen MA, DeMaere MZ, Kyrpides NC, Tringe SG, Woyke T, Cavicchioli R. 2014. Microbial ecology of an Antarctic hypersaline lake: genomic assessment of ecophysiology among dominant haloarchaea. ISME J 8:1645–1658. doi:10.1038/ismej.2014.18 24553470 PMC4817606

[28] DeMaere MZ, Williams TJ, Allen MA, Brown MV, Gibson JAE, Rich J, Lauro FM, Dyall-Smith M, Davenport KW, Woyke T, Kyrpides NC, Tringe SG, Cavicchioli R. 2013. High level of intergenera gene exchange shapes the evolution of haloarchaea in an isolated Antarctic lake. Proc Natl Acad Sci USA 110:16939–16944. doi:10.1073/pnas.1307090110 24082106 PMC3801024

[29] Oren A. 2024. Novel insights into the diversity of halophilic microorganisms and their functioning in hypersaline ecosystems. NPJ Biodivers 3:18. doi:10.1038/s44185-024-00050-w 39242694 PMC11332174

[30] Hoops S, Sahle S, Gauges R, Lee C, Pahle J, Simus N, Singhal M, Xu L, Mendes P, Kummer U. 2006. COPASI--a COmplex PAthway SImulator. Bioinformatics 22:3067–3074. doi:10.1093/bioinformatics/btl485 17032683

